# Survival and hormone production of isolated mouse follicles in three-dimensional artificial scaffolds after stimulation with bpV(HOpic)

**DOI:** 10.1007/s00404-024-07419-z

**Published:** 2024-03-12

**Authors:** Philip Keckstein, Ralf Dittrich, Nathalie Bleisinger, Inge Hoffmann, Matthias W. Beckmann, Albrecht Gebhardt, Benjamin Schmid, Simon Keckstein

**Affiliations:** 1 Department of Gynecology and Obstetrics, Erlangen University Hospital, Friedrich-Alexander-University of Erlangen–Nürnberg, Comprehensive Cancer Center ER-EMN, Erlangen, Germany; 2 Department of Statistics, University of Klagenfurt, Klagenfurt, Austria; 3 Optical Imaging Center Erlangen (OICE), University of Erlangen–Nürnberg, Erlangen, Germany; 4 Department of Obstetrics and Gynecology, LMU University Hospital, LMU Munich, Munich, Germany

**Keywords:** bpV(HOpic), Ovarian follicle, Artificial ovary

## Abstract

**Purpose:**

To preserve fertility before gonadotoxic therapy, ovarian tissue can be removed, cryopreserved, and transplanted back again after treatment. An alternative is the artificial ovary, in which the ovarian follicles are extracted from the tissue, which reduces the risk of reimplantation of potentially remaining malignant cells. The PTEN inhibitor bpV(HOpic) has been shown to activate human, bovine and alpacas ovarian follicles, and it is therefore considered a promising substance for developing the artificial ovary. The purpose of this study was to examine the impact of different scaffolds and the vanadate derivative bpV(HOpic) on mice follicle survival and hormone secretion over 10 days.

**Methods:**

A comparative analysis was performed, studying the survival rates (SR) of isolated mice follicle in four different groups that differed either in the scaffold (polycaprolactone scaffold versus polyethylene terephthalate membrane) or in the medium—bpV(HOpic) versus control medium. The observation period of the follicles was 10 days. On days 2, 6, and 10, the viability and morphology of the follicles were checked using fluorescence or confocal microscopy. Furthermore, hormone levels of estrogen (pmol/L) and progesterone (nmol/L) were determined.

**Results:**

When comparing the SR of follicles among the four groups, it was observed that on day 6, the study groups utilizing the polycaprolactone scaffold with bpV(HOpic) in the medium (SR: 0.48 ± 0.18; *p* = 0.004) or functionalized in the scaffold (SR: 0.50 ± 0.20; *p* = 0.003) exhibited significantly higher survival rates compared to the group using only the polyethylene terephthalate membrane (SR: 0). On day 10, a significantly higher survival rate was only noted when comparing the polycaprolactone scaffold with bpV(HOpic) in the medium to the polyethylene terephthalate membrane group (SR: 0.38 ± 0.20 versus 0; *p* = 0.007). Higher levels of progesterone were only significantly associated with better survival rates in the group with the polycaprolactone scaffold functionalized with bpV(HOpic) (*p* = 0.017).

**Conclusion:**

This study demonstrates that three-dimensional polycaprolactone scaffolds improve the survival rates of isolated mice follicles in comparison with a conventional polyethylene terephthalate membrane. The survival rates slightly improve with added bpV(HOpic). Furthermore, higher rates of progesterone were also partly associated with improved survival.

## What does this study add to the clinical work?

**Table:** 

The importance of fertility preservation measures is increasing in our society, therefor it is crucial to explore innovative methods. In this work, we further investigated the approach of in vitro maturation of primordial follicles and the development of an artificial ovary.	

## Introduction

The need for fertility preservation measures has increased dramatically in recent years. The reasons for this may be oncological or benign diseases, on the one hand, as well as patients’ personal motives on the other [1].

Survival rates for pediatric and adult cancer patients have also increased rapidly across Europe in recent decades, due to improved treatments and supportive care [2, 3]. However, the ovaries are highly sensitive to cytotoxic drugs and radiation, so that cancer survivors are at considerable risk of premature ovarian insufficiency as a result of oncological treatment. These women may face the challenge of undergoing hormone replacement therapy for extended periods, as well as the distressing prospect of infertility [4].

The provision of fertility protection methods is regarded as essential in national and international guidelines, and several options are available to help these patients [5]. In addition to the techniques for cryopreserving embryos and mature oocytes, new fertility preservation methods have emerged in recent years. These include the cryopreservation of ovarian tissue followed by reimplantation at a later stage, with reported successful live birth rates of approximately 30% [6–9]. It has the added advantage that women benefit from residual hormonal activity in the ovarian tissue for several years [10–12]. For women who are suffering from a systemic oncological disease such as acute leukemia, this method may involve a risk of reimplantation of malignant cells after the treatment has been completed [13–15]. Innovative approaches, such as the in vitro maturation of primordial follicles or the development of an artificial ovary, are therefore necessary to give also these patients the change of having a child in the future.

In the current study, our primary objective was to assess and compare the impact of different scaffolds and the vanadate derivative dipotassium bisperoxo(5-hydroxypyridine-2-carboxyl)oxovanadate, referred to as bpV(HOpic), on follicle survival, as well as estrogen and progesterone secretion, over a 10-day observation period. Previous studies have demonstrated the ability of the PTEN inhibitor bpV(HOpic) to activate ovarian follicles in humans, pigs, and alpacas [16–18]. Despite its promise in enhancing follicle maturation, investigations into its influence on follicle growth within three-dimensional scaffolds are lacking.

Two-dimensional and three-dimensional follicle culture systems have been investigated previously as a basis for an artificial ovary [19]. While two-dimensional culture systems have proved successful in experimental settings, more recent studies show that three-dimensional culture systems allow better mimicry of the physiological structure of the ovary, preserving the follicular architecture and the interplay between germ cells and somatic cells [20–22]. In recent years, various types of three-dimensional scaffolds, including gelatin or fibrin-based structures, decellularized ovarian tissues, and biomimetic hydrogels, have been developed [23–27].

Scaffolds constructed by electrospinning were used in three of the four experimental groups. In the first group, bpV(HOpic) was added to the medium, while in the second group, the scaffold itself was functionalized with bpV(HOpic). The third group served as a control for the electrospun scaffold, using a standard medium. The fourth group’s scaffold was a commercially available insert made of a polyethylene terephthalate (PET) membrane with 0.4-μm pores without a fibrillar structure and served as a structural control [28].

## Material and methods

### Study groups

The study investigated the survival rate of mice follicles in four different groups, in which the scaffold or medium differed:

- Group 1: Polycaprolactone (PCL) scaffold, with 1 µM bpV(HOpic) (Sigma-Aldrich Chemicals, St. Louis, Missouri, USA) in the medium (hereafter abbreviated as HM)
- Group 2: PCL scaffold functionalized with 1 µM bpV(HOpic), with normal medium (further details are described in *2.3 Cultivation of the follicles and viability testing*) (hereafter abbreviated as HS)
- Group 3: PCL scaffold with normal medium (hereafter abbreviated as CS)
- Group 4: PET membrane (Millicell Cell Culture Insert, 30 m, 0.4 µm; Sigma-Aldrich Chemicals, St. Louis, Missouri, USA), with normal medium as control (hereafter abbreviated as CP).

### Polycaprolactone scaffolds and polyethylene terephthalate membrane

Our group’s previously published methodology was used to create the PCL scaffolds [29–31]. PCL is a biodegradable polyester that exhibits a low rate of degradation in an aqueous environment, with harmless by-products, and its properties can be modulated by mixing it with other polymers [29].

In summary, PCL (80 kDa) obtained from Sigma-Aldrich was dissolved in glacial acetic acid (20%w/v). Electrospinning was performed with different solutions employing a commercial device (EC-CLI, IME Medical Electrospinning) equipped with a gasshield accessory to optimize the instability region. The process took place in a climate chamber set at 25 ˚C with 25% relative humidity. An applied voltage of 15 kV was used, and the distance between the needle tip (23G diameter) and the fiber collector was maintained at 11 cm. The solutions were extruded at a flow rate of 0.4 mL/h. To create patterned mats with macropores, a specific collector consisting of a grid (with a space of 500 μm between struts) was employed. The grid was shaped into a circle with a diameter of 2.5 cm, producing samples suitable for multiwell cell culture. The electrospinning process lasted for 5 min [29].

In the process of functionalization the PCL electrospun scaffolds with bpV(HOpic), samples were placed in a 24-multiwell plate and disinfected under UV light for 1 h. A 0.01 M NaOH solution was applied (1 mL per well) for 20 min at 37 °C. After removal of the solution, samples were washed twice with PBS (Phosphate Buffered Saline) for 5 min each. Then, 1 mL of 1 µM bpV(HOpic) solution was added to each well and removed after 24 h. Then followed by another PBS wash to remove excess bpV(HOpic). Samples were then ready for seeding. In Group 1, the medium with 1 μm bpv (HOpic) was replaced after 24 h with normal medium.

The PET membrane is used for cell cultivation and can be purchased commercially, in contrast to the PCL scaffolds. The PET membrane is flat and does not have a fibrillar structure, in contrast to the PCL scaffold.

### Cultivation of the follicles and viability testing

The observation period was 10 days. On days 2, 6, and 10, the viability and morphology of the follicles was checked using fluorescence microscopy or confocal microscopy. The experiments were repeated four times for each culture condition. The follicles were cultured in groups within each well, with possible variability in the number of follicles per group (Table 1). However, the numbers of follicles per experimental series and group remained constant. The hormones progesterone and estrogen were measured from the culture medium every second day, as well as at the end of the experiment, using automated electrochemiluminescence technology for immunoassay analysis (Cobas e601; Roche Diagnostics Deutschland GmbH, Mannheim, Germany).

**Table 1:** The table shows the total number of follicles used per experimental group and day of examination of the follicle viability

	Day 2	Day 6	Day 10
HM	95	115	115
HS	96	115	115
CS	95	115	115
CP	120	120	120

The mouse has proved to be a suitable model for studying human reproductive biology [32]. Mice (C57/6N; Janvier Labs, Le Genest-Saint-Isle, France) were obtained from the Biotechnologisches Entwicklungslabor BTE (Friedrich-Alexander-University of Erlangen–Nürnberg, Germany). The mice used for follicle isolation were approximately 6 weeks old. Whole mice ovaries were excised from the fat tissue and washed with Dulbecco’s phosphate-buffered saline (DPBS) (D8661; Sigma-Aldrich Chemicals, St. Louis, Missouri, USA). The ovaries were then transferred into a 50-mL Falcon tube (Corning Inc., Corning, New York, USA) containing 3.0 mL lysis buffer, consisting of 3.0 mg collagenase (C2674; Sigma-Aldrich, St. Louis, Missouri, USA) in DPBS (the final concentration is 1.5 mg/mL). To activate enzymatic digestion, the tube was incubated in a water bath at 37 °C for 30 min. During this period, the Falcon tube was gently shaken for about 20–30 s at 10-min intervals. To stop the enzymatic process, 5 mL of cold DPBS was added into the tube. Before the contents were moved into a Petri dish (Nunc IVF Petri dishes, 90 × 17 mm; ThermoFisher Scientific, Waltham, Massachusetts, USA), the tube was shaken again to detach further follicles from the tissue residues. Follicle collection was carried out under an inverted microscope (CKX53; Olympus, Shinjuku, Tokyo, Japan) under 250× magnification. We attempted to culture exclusively primordial follicles, which measured approximately 50–80 µm in size. After the follicles had been collected, they were washed three times in sterile DPBS and gathered in a droplet of culture medium. For each scaffold, a separate droplet was prepared in a Petri dish (Nunc IVF Petri dishes, 60 × 15 mm; ThermoFisher Scientific, Waltham, Massachusetts, USA). The number of follicles collected was checked twice before seeding.

The electrospun scaffolds and the PET membrane were placed in 24-well plates (Greiner CELLSTAR®; Sigma-Aldrich, St. Louis, Missouri, USA). Before the follicles were seeded onto the scaffolds, they were disinfected using ultraviolet light irradiation (UV Transilluminator; Bachofer Laboratoriumsgeräte, Reutlingen, Germany) for 60 min.

During the collection and seeding process, a Stripper micropipettor with a 175-μm Stripper tip (CooperSurgical, Inc., Trumbull, Connecticut, USA) was used. After collection and pipetting of the correct number of follicles onto the scaffold, the Stripper tip was checked again for any remaining follicles.

**Fig. 1:**
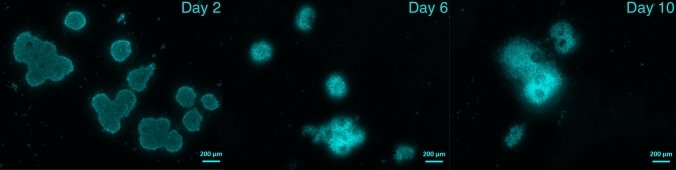
The morphology of ovarian follicles was categorized on days 2, 6, and 10 of culture using 4',6-diamidino-2-phenylindole (DAPI) staining. Day 2: Follicles exhibited a spherical shape with a complete granulosa cell layer. Day 6: Follicles formed clusters and gradually acquired an irregular shape. Day 10: Follicles remained partly viable. Scale bars represent 200 µm

**Fig. 2:**
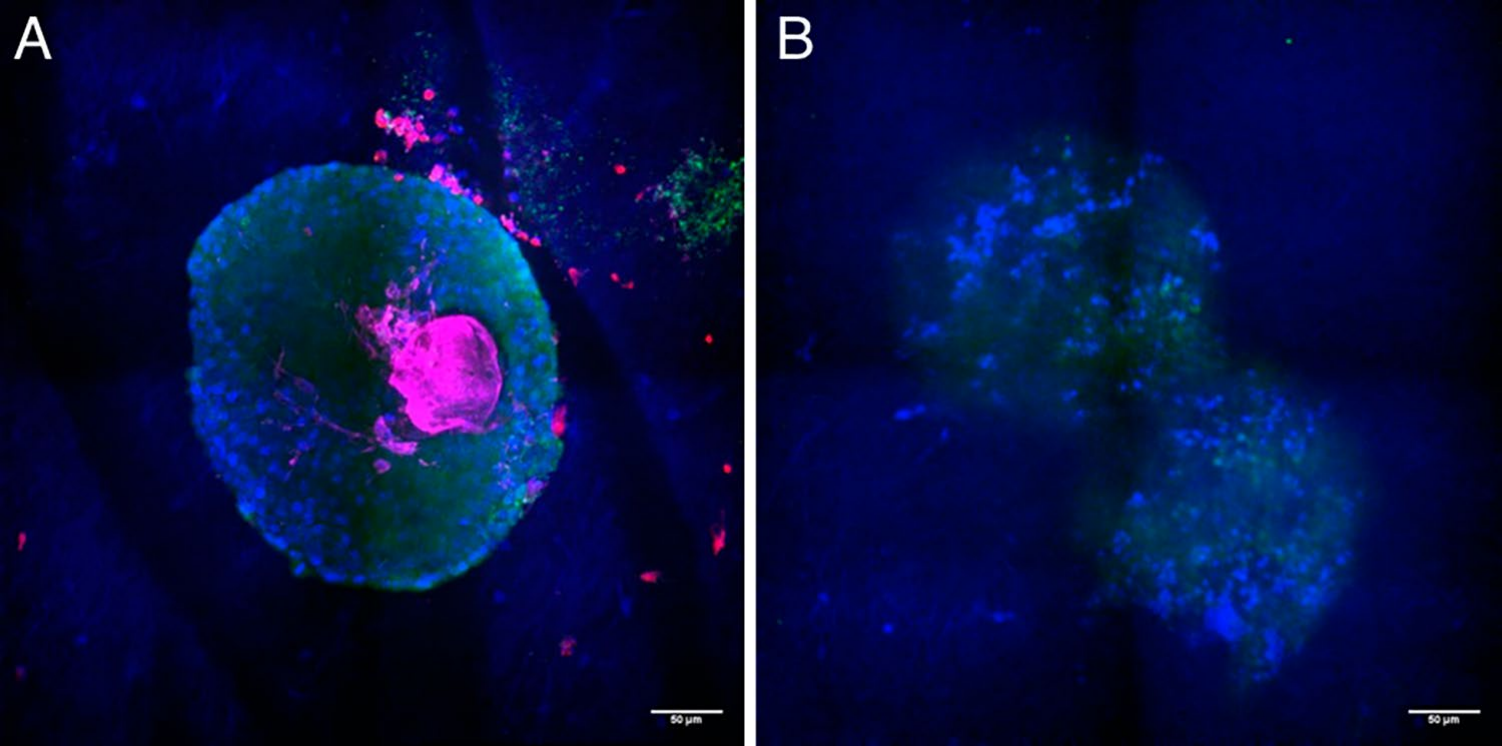
Confocal microscopy images of the follicles on Day 2 inside the PET-Membrane (**A**) and Functionalized PCL-Scaffold (**B**). **A** Follicles show no contact or interaction to the mesh of the PET-Membrane. **B**: Shows the follicle on Day 2 deep inside the Functionalized PCL scaffold. Green points show living cells, the blue fibers represent the electrospun mesh with the embedded follicles and pink represent the SiR-F-actin staining. Scale bar 50 μm

Each scaffold with seeded follicles was cultivated in 1.2 mL of a modified version of the serum-free medium used by Telfer et al. [33]: McCoy’s 5a medium (LifeTechnologies, Carlsbad, California, USA) supplemented with 20 mM HEPES buffer (ThermoFisher Scientific, Waltham, Massachusetts, USA), 0.1% bovine serum albumin (BSA) (CarlRoth, Karlsruhe, Germany), 3 mM l-glutamine (ThermoFisher Scientific, Waltham, Massachusetts, USA), Fungizone (final amphotericin B concentration 2.5 μg/mL; ThermoFisher Scientific, Waltham, Massachusetts, USA), 0.1 mg/mL each of penicillin and streptomycin (Sigma-Aldrich, St. Louis, Missouri, USA), insulin–transferrin–sodium selenite (ITS) solution (final concentrations: selenium 4 ng/mL, transferrin 4.4 μg/mL, and insulin 8 μg/mL; Sigma-Aldrich, St. Louis, Missouri, USA), 50 μg/mL ascorbic acid (Sigma-Aldrich, St. Louis, Missouri, USA), and 0.272 IU recombinant follicle-stimulating hormone (rFSH) (Gonal-f; Merck, Darmstadt, Germany). The incubation time was 10 days at 37 °C in humidified air with 5% CO_2_. The natural progression of follicle growth in mice, from preantral to mature follicle size, typically spans a period of 10–12 days [34]. Half of the medium was replaced every second day. The viability of the follicles was assessed using LIVE/DEAD assays (ThermoFisher Scientific, Waltham, Massachusetts, USA), in which calcein retained from living cells produces green fluorescence and ethidium homodimer-1 interacts, producing red fluorescence. SiR-F-actin, Hoechst-33342 and 4′,6-diamidino-2-phenylindole (DAPI) staining (shown in Fig. 1) (ThermoFisher Scientific, Waltham, Massachusetts, USA) was also used. The samples were evaluated using a fluorescence microscope (Axio Scope A1; Zeiss, Jena, Germany). Confocal microscopy images of the different scaffolds are shown in Fig. 2. The attachment of the follicles to the scaffold was tested by rinsing the scaffold’s surface with a pipette containing medium. Unattached or nonviable follicles were subsequently washed away.

### Measurement of hormone concentrations

Concentrations of estradiol (measured in pmol/L) and progesterone (measured in nmol/L) in culture media from the isolated follicles were determined with immunoassays (Roche®; Roche Diagnostics Deutschland GmbH, Mannheim, Germany).

### Statistical analysis

Concerning the survival rate and hormone values, the data was initially analyzed using a classic analysis of variance (ANOVA) setting with post-hoc t tests. Following data reorganization, a generalized linear model was employed for further analysis (as described in more detail in the “Results” section). *p* values below 0.05 were considered statistically significant. The statistical programming environment R, version 4.0.2, was used for processing and analysis of the data.

## Results

### Survival rate

Table 2 presents means and standard deviations for survival rates in the four experimental groups on the respective examination days.

**Table 2:** The table presents the survival rate with the corresponding standard deviation for each group on each day

	HM	HS	CS	CP
Day 2	0.54 ± 0.48	0.60 ± 0.08	0.44 ± 0.17	0.16 ± 0.03
Day 6	0.48 ± 0.18	0.50 ± 0.20	0.34 ± 0.15	0
Day 10	0.38 ± 0.20	0.26 ± 0.11	0.21 ± 0.11	0

**Fig. 3:**
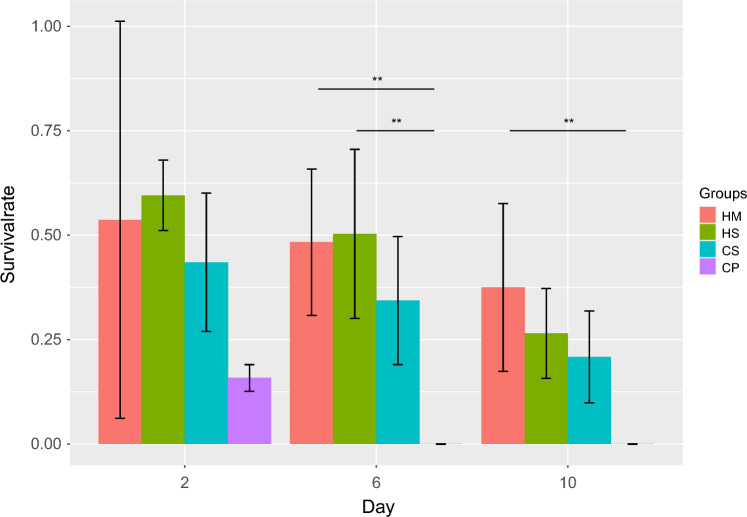
The figure represents the mean survival of follicles of each group on each examination day along with the standard deviation. Significant differences in the post-hoc *t* tests are marked with an asterisk (*p* < 0.01: **)

The data collected in the experiments were first analyzed using a classic analysis of variance (ANOVA) setting with post-hoc t tests to assess whether the number of surviving follicles was dependent on the treatment applied. On day 2, significant differences among the four experimental groups were already observed in the ANOVA analysis: F(1,4) = 5.229, *p* = 0.038. However, the post-hoc tests did not reveal significant results. On day 6, highly significant differences among the four groups were observed in the ANOVA analysis: F(1,14) = 17.38, *p* < 0.001. The post-hoc tests showed significant differences when groups HM and HS were compared with CP. On day 10, highly significant differences continued to be observed in the ANOVA analysis: F(1,14) = 18.62, *p* < 0.001. The post-hoc tests also revealed significant differences when groups HM and CP were compared. The results of the post-hoc t test are shown in Table 3 and Fig. 3.

**Table 3:** The table presents the results of the post-hoc tests of the ANOVA

	Day 2			Day 6			Day 10		
	HM	HS	CS	HM	HS	CS	HM	HS	CS
HS	1.00			1.00			1.00		
CS	1.00	1.00		1.00	1.00		0.522		
CP	0.35	0.19	0.91	0.004**	0.003**	0.050	0.007**	0.071	0.228

Significant results are marked with an asterisk (*p* < 0.01: **)

### Logistic regression model for survival rate

**Fig. 4:**
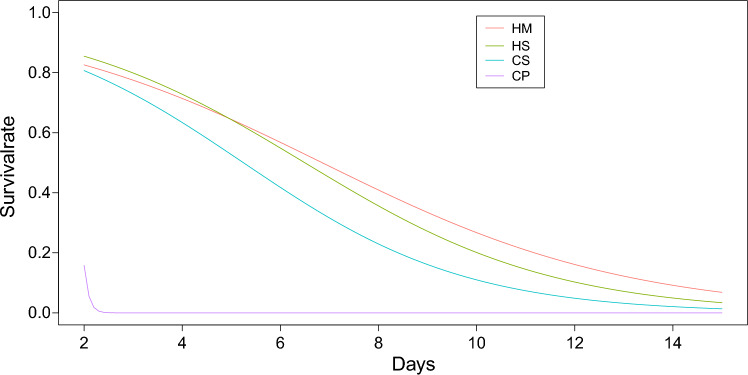
The figure shows the survival probabilities of four study groups over time, based on the logistic regression models. The y-axis represents the calculated survival rate, with 1.0 indicating 100% vital follicles, and a value of 0 signifying the absence of viable follicles

The data were now slightly reorganized in order to model the long-term behavior involved in follicle survival. From this point on, all follicles from one well were treated as individuals—in most cases, 30 per well. This represents 1336 individuals being observed during the whole experiment across all the different treatment groups. Whether or not they survived up to a specific certain day was now encoded as either 0 or 1. This makes it possible to model the probability of survival of these individual follicles using a generalized linear model, specifically a logistic regression model, via:

$${\text{P}}\left({\text{Survival}}\right)=\frac{1}{1-{{\text{e}}}^{{\text{f}}({\text{D}})}}$$

with a linear regression function ${\text{f}}\left({\text{D}}\right)={\mathrm{\vartheta }}_{0}+{\mathrm{\vartheta }}_{1}{\text{D}}$ in the above logistic function, where ${\text{D}}$ denotes days from the start of the experiment. This logistic regression model was applied to all four study groups, including the control group, resulting in the prediction of survival probabilities for all four cases shown in Fig. 4. The three groups HM, HS, and CS clearly outperformed the control group CP.

### Logistic regression model for survival rate and hormone levels

More detailed analysis was applied to these three treatments, taking progesterone and estradiol measurements into account. The hormone values for each of the respective groups are shown in Table 4. Initially, also an ANOVA was performed with subsequent post-hoc t-tests. In this analysis, no significant differences were observed in the measurements of estrogen and progesterone when comparing the groups with each other.

**Table 4:** Mean values with standard deviation for estrogen (pmol/L) and progesterone (nmol/L) for each group on each day

	HM	HS	CS
Day 2			
Estradiol	84.82 ± 9.23	74.02 ± 7.79	85.78 ± 11.07
Progesterone	0.39 ± 0.19	0.25 ± 0.09	0.44 ± 0.34
Day 6			
Estradiol	81.24 ± 25.19	82.70 ± 14.48	105.73 ± 25.36
Progesterone	0.60 ± 0.33	0.62 ± 0.35	1.00 ± 0.68
Day 10			
Estradiol	77.98 ± 10.58	85.62 ± 16.07	81.06 ± 21.23
Progesterone	0.49 ± 0.25	0.66 ± 0.45	0.68 ± 0.62

To individually investigate the influence of the hormones on each group, the regression function ${\text{f}}\left({\text{D}}\right)={\mathrm{\vartheta }}_{0}+{\mathrm{\vartheta }}_{1}{\text{D}}+{\mathrm{\vartheta }}_{2}{\text{P}}+{\mathrm{\vartheta }}_{3}{\text{E}}$ in the denominator of the above setup of the logistic regression model equation was used. Here ${\text{P}}$ denotes the progesterone concentration and ${\text{E}}$ the estradiol concentration. The hormone concentrations in each experimental group were individually examined relative to each day. The results are shown in Table 5.

**Table 5:** The Table displays the results of the generalized linear model described above, considering measurements of the respective day, progesterone (PROG), and estradiol (E2)

	Estimate	Standard error	*z* score	*p* value
HM				
Day	− 0.1425	0.0399	− 3.569	< 0.001***
Estradiol	− 0.0040	0.0078	− 0.523	0.600
Progesterone	0.5373	0.5044	1.065	0.286
HS				
Day	− 0.2137	0.0514	− 4.157	< 0.001***
Estradiol	− 0.0102	0.0104	− 0.974	0.330
Progesterone	0.9887	0.4156	2.379	0.017*
CS				
Day	− 0.1065	0.0431	− 2.469	0.013*
Estradiol	0.0022	0.0073	0.307	0.759
Progesterone	0.1632	0.2836	0.575	0.565

It presents the estimated regression parameters, standard errors, *z*-values, and *p* values. Significant results are indicated by an asterisk (*p* < 0.05: *; *p* < 0.001: ***)

**Fig. 5:**
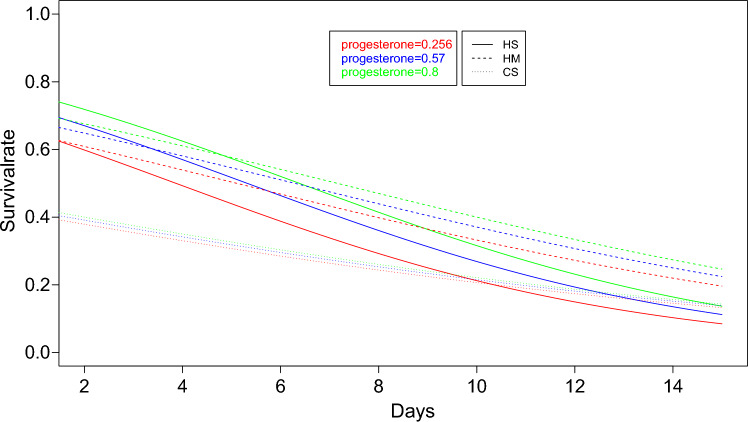
The figure shows the survival probabilities of the three study groups over time, based on the logistic regression models and their respective progesterone levels, with estrogen fixed at its median value (80.53)

The data suggest that follicle survival correlates more closely with progesterone and less well with estrogen. Figure 5 thus indicates the dependency of progesterone on follicle survival when estrogen remains fixed at its median value and progesterone is fixed at the lower quartile, median, and upper quartile.

## Discussion

The aim of this study was to compare the different scaffolds with each other, and also to observe any potential stimulating effect of bpV(HOpic) in relation to the survival of the follicles and the secretion of estrogen and progesterone.

In the comparison of the SR of follicles across the four groups, the results showed on day 6, that the study groups HS (SR: 0.48 ± 0.18; *p* = 0.004) and HM (SR: 0.50 ± 0.20; *p* = 0.003) displayed significantly higher survival rates compared to the CP group (SR: 0). On day 10, a significantly higher survival rate was only noted when comparing the HM to the CP group (SR: 0.38 ± 0.20 versus 0; *p* = 0.007). Higher levels of progesterone were only significantly associated with better survival rates in the HS group (*p* = 0.017).

The first control group in the study represented a commercially available insert, which has a PET membrane with 0.4-μm pores. This is used for cell culture because the pores allow the medium to be exchanged and support the cells. The scaffold’s surface is flat and does not have a fibrillar structure, which is why it was used as a structural control. In theory, ovarian follicles cultivated with this type of membrane are incapable of establishing sufficient or appropriate contact with the fibers [23]. This is in line with the study’s results, which demonstrated that the follicles tended to be unable to maintain their morphology, ultimately leading to a decreased survival rate.

The PCL scaffold served as a second structural control and as a basic scaffold. Ovarian follicles interact with the material as well as the three-dimensional design of the fibers, and they remain vital in the supporting scaffolds [29, 31]. The follicles’ morphology can therefore also be maintained in the scaffolds, and the materials as well as the three-dimensional structure play an important role in follicle survival. Microscopic imaging also showed interaction between the follicles and the PCL scaffold, which was observable in all three study groups. The follicles established contact with the fibers of the support scaffolds, as shown by their higher survival rates in comparison with the PET membrane. All of the PCL scaffold groups in the experiments showed larger numbers of vital follicles and morphologically intact follicles in comparison with the PET membrane.

Studies have identified the phosphoinositide 3‐kinase‐protein kinase B (PI3K‐Akt) as a crucial enzyme for follicle activation [35]. The activation of follicles through this pathway is effectively inhibited by the phosphatase and tensin homolog (PTEN), thus preventing premature follicular development under physiological conditions John, Gallardo [35]. BpV(HOpic) is known to be a pharmacological inhibitor of PTEN and its effect on human and porcine follicles have been investigated in previous studies, which showed that it had a significant impact on the development and growth of human and porcine ovarian follicles [25, 27, 33]. As the influence of bpV(HOpic) has previously only been investigated in unprocessed ovarian tissue, the aim was to investigate its effect on isolated mice follicles on artificial ovarian scaffolds.

Li et al. [36] demonstrated that bpV(HOpic) enhances follicular growth and oocyte development. These results were confirmed by McLaughlin et al. [18] in relation to larger numbers of activated primordial and secondary follicles. However, the isolated secondary follicles that appeared to be vital were unable to develop and survive as well as expected.

When the four groups in the present study were compared in the ANOVA, only those groups treated with bpV(HOpic) showed significantly higher survival rates in comparison with the group with the PET membrane: the groups with bpV(HOpic) in the medium on days 6 and 10, and the group with the bpV(HOpic)-functionalized scaffold on day 6. Although slightly better survival rates were observed in both bpV(HOpic)-treated groups in comparison with the PCL control group, the differences did not reach statistical significance. Thus, bpV(HOpic) may have an influence on survival, the choice of the scaffold material has a more significant impact on the probability of survival. However, Maidarti et al. noted increased DNA damage and reduced DNA repair capacity in bovine ovarian follicles after exposure to different doses (1 and 10 μM) of bpV(HOpic) [34]. This is also a major limitation in the present study, as it was able to demonstrate improved follicle survival with bpV(HOpic), but not to reach any conclusions about the functionality or health of the follicles or potential DNA damage.

This constitutes a notable limitation in the current study, as it does not provide insights into the overall health of the follicles or the potential of a DNA damage. Although *PTEN* inhibitors have been used effectively to restore fertility in patients with primary ovarian insufficiency, by stimulating primordial follicles in vitro in fragmented tissue that was then transplanted back into the patients [37–39], the question of whether there is potential DNA damage certainly needs to be further investigated.

This study also investigated potential follicular secretion of estrogen and progesterone over time. In principle, increasingly substantial hormone production is observed in advanced antral follicles. In animal experimental studies, however, the capacity of follicles to produce estrogen and progesterone has already been demonstrated at significantly earlier stages [40]. In the present study, a significant positive correlation was observed between the overall survival of follicles and the progesterone concentrations measured, particularly in the group with the scaffold functionalized with bpV(HOpic). This finding is consistent with previous research results. On the one hand, it can be assumed that when there are more viable follicles, larger amounts of hormone will consequently be produced. On the other hand, increased hormone concentrations might also be caused by the activation of follicles into more advanced stages induced by bpV(HOpic).

## Conclusion

In conclusion, the results of this study confirm that follicles have a significant survival advantage when cultured in a three-dimensional scaffold in comparison with conventional membranes. The addition of bpV(HOpic) further improves the survival rates, though not significantly. Higher progesterone levels partly correlate with better survival rates. Currently, it is not possible to draw definitive conclusions regarding the extent to which isolated follicles develop under the influence of bpV(HOpic). This aspect will require further intensive investigation in the future.

## Data Availability

Data are available upon reasonable request.
